# The cost-effectiveness of implementing HPV testing for cervical cancer screening in El Salvador

**DOI:** 10.1002/ijgo.12773

**Published:** 2019-01-31

**Authors:** Nicole G. Campos, Mauricio Maza, Karla Alfaro, Julia C. Gage, Philip E. Castle, Juan C. Felix, Rachel Masch, Miriam Cremer, Jane J. Kim

**Affiliations:** 1 Department of Health Policy and Management, Harvard T.H. Chan School of Public Health, Center for Health Decision Science, Boston, MA, USA; 2 Basic Health International, Colonia San Francisco, San Salvador, El Salvador; 3 Department of Cancer Epidemiology and Genetics, National Cancer Institute, Rockville, MD, USA; 4 Albert Einstein College of Medicine, Bronx, New York, NY, USA; 5 Department of Pathology, Medical College of Wisconsin, Milwaukee, WI, USA; 6 Icahn School of Medicine at Mount Sinai, New York, NY, USA; 7 Obstetrics, Gynecology & Women's Health Institute, Cleveland Clinic Lerner College of Medicine, Cleveland, OH, USA

**Keywords:** Cancer screening, Cost-effectiveness analysis, Decision analysis, El Salvador, HPV DNA tests, Human papillomavirus (HPV), Mathematical model, Uterine cervical neoplasms

## Abstract

**Objective:**

To assess the cost-effectiveness of HPV-based screening and management algorithms for HPV-positive women in phase 2 of the Cervical Cancer Prevention in El Salvador (CAPE) demonstration, relative to the status quo of Pap-based screening.

**Methods:**

Data from phase 2 of the CAPE demonstration (n=8000 women) were used to inform a mathematical model of HPV infection and cervical cancer. The model was used to project the lifetime health and economic outcomes of HPV testing every 5 years (age 30–65 years), with referral to colposcopy for HPV-positive women; HPV testing every 5 years (age 30-65 years), with immediate cryotherapy for eligible HPV-positive women; and Pap testing every 2 years (age 20–65 years), with referral to colposcopy for Pap-positive women.

**Results:**

Despite slight decreases in the proportion of HPV-positive women who received treatment relative to phase 1, the health impact of screening in phase 2 remained stable, reducing cancer risk by 58.5%. As in phase 1, HPV testing followed by cryotherapy for eligible HPV-positive women remained the least costly and most effective strategy (US$490 per year of life saved).

**Conclusion:**

HPV-based screening followed by immediate cryotherapy in all eligible women would be very cost-effective in El Salvador.

## 1 INTRODUCTION

Cervical cancer is the leading cause of cancer death among women in El Salvador.^1^ Screening to detect precancerous lesions caused by persistent infection with oncogenic human papillomavirus (HPV) can prevent cervical cancer. Yet conventional screening with Pap testing has faced challenges in El Salvador and other low-resource settings due to low population coverage, the need for frequent screening, and insufficient navigation to treatment for women who are screen positive.

WHO recommends screening with HPV DNA testing where resources are available.^2^ Following a positive HPV test, WHO has endorsed management strategies involving either immediate treatment with cryotherapy, referral to colposcopy, or triage testing with visual inspection with acetic acid (VIA), in which only women who are both HPV positive and VIA positive are referred for treatment. Further data on the cost-effectiveness of these management strategies are needed.

In 2012, the Cervical Cancer Prevention in El Salvador (CAPE) project was launched to assess the feasibility and cost-effectiveness of incorporating low-cost HPV testing into the national cervical cancer screening program. The CAPE project is a demonstration project in three phases, conducted by the Salvadoran Ministry of Health (MINSAL) with technical support from the non-profit organization Basic Health International. In phase 1—a pilot study of 2000 women aged 30–49 years screened at four health centers in the Paracentral region—women who tested positive for HPV received one of two management algorithms: colposcopy management (referral to colposcopy followed by treatment for women with cervical intraepithelial neoplasia), or screen and treat (immediate treatment for all eligible women).^3^ Phase 2 scaled up HPV testing to 8000 women at eight health centers in order to compare the two management algorithms in a larger and more diverse screening population.^4^

In phase 1, more women received recommended follow-up in the screen and treat cohort than in the colposcopy management cohort, and screen and treat was found to be very cost-effective for management of HPV-positive women in El Salvador.^3^,^5^ The objective of the present analysis was to use data from CAPE phase 2 to evaluate the cost-effectiveness of the colposcopy management and screen and treat management algorithms (relative to Pap-based screening) as implementation and scale-up continues in the public sector.

## 2 MATERIALS AND METHODS

The present study was conducted using an individual-based Monte Carlo simulation model of the natural history of HPV and cervical cancer,^6,7^ as in phase 1. The model projects the lifetime health and economic outcomes associated with each screening strategy. As described in previous publications, individual girls enter the simulation model at age 9 years and transition between health states (including type-specific HPV infection status, histologic grade of precancer [CIN2 or 3], and stage of cancer) each month until death. Monthly transition probabilities may vary by age, HPV type, duration of infection or lesion status, and prior HPV infection. Death from all causes can occur from any health state, and excess mortality from cervical cancer can occur after its onset, depending upon the stage of cancer. The model tracks disease progression and regression, screening and treatment events, and healthcare costs over the lifetime of each woman. These outcomes are then aggregated over the population and used for analysis.^6,7^

Details of the model parameterization and calibration process have been described elsewhere.^6-8^ In brief, baseline parameter values were established for the natural history component of the model using longitudinal data for age- and type-specific HPV incidence, as well as type- specific and time-dependent rates of HPV clearance and progression.^9-13^ To reflect differences in HPV incidence and burden between settings, in addition to uncertainty in the degree of natural immunity following initial infection and in progression and regression of precancer, plausible ranges were set around these input parameter values. Repeated natural history model simulations selected a single random value from the range for each uncertain parameter to form a unique natural history input parameter set. A goodness-of-fit score was then computed for each unique set by summing the log-likelihood of model-projected outcomes to represent the quality of fit to epidemiologic data (i.e., calibration targets), including the age-specific prevalence of oncogenic HPV among women aged 30–49 years in phase 2 of the CAPE project,^4^ prevalence of HPV genotypes 16 and 18 in cervical cancer in South and Central America,^14^ and age-specific cervical cancer incidence in El Salvador.^1^ The 50 top good-fitting input parameter sets were selected for use in cost-effectiveness analysis, as a form of probabilistic sensitivity analysis (Figures S1–S3). Results were reported as the mean and range of outcomes across these top 50 parameter sets.

As in the phase 1 cost-effectiveness analysis, Pap testing was compared with two HPV screening and management algorithms (colposcopy management and screen and treat) for women who tested positive for HPV. Following an initial screening visit at the clinic, women were scheduled to return for results. Women in the colposcopy management cohort who screened positive were then scheduled to receive colposcopy at the designated hospital, whereas women in the screen and treat cohort received visual assessment to determine eligibility for immediate cryotherapy (with ineligible women referred to colposcopy).

It was assumed that (1) the initial screening populations were identical for each strategy; (2) the proportion of women who attended visits to receive results, cryotherapy, colposcopy, and treatment were based on phase 2 data from the relevant cohort (colposcopy management or screen and treat) and complied with recommended follow-up within 6 months (Table 1)^4^; and (3) compliance in the Pap strategy (which was not examined in the CAPE project) would be the same as in colposcopy management, as women were referred to colposcopy following a positive Pap result.

**TABLE 1:** Baseline values and ranges for model variables.^a^

Variable [reference]	Baseline value	Sensitivity analyses
Population coverage of screening program	80%	40%, 60%
Results visit compliance^3,4b^		
CM and Pap	Phase 1: 100% Phase 2: 100%	Phase 2: 90%
ST	Phase 1: 100% Phase 2: 100%	Phase 2: 90%
Cryotherapy compliance^3,4b^		
CM and Pap	NA	NA
ST	Phase 1: 100% Phase 2: 97.3%	Phase 2: 95.0%
Colposcopy compliance^3,4b,c^		
CM and Pap	Phase 1: 88.2% Phase 2: 79.1%	Phase 2: 75.0%
ST	Phase 1: 100% Phase 2: 53.6%	Phase 2: 39.7%
Treatment compliance^3,4b,c^		
CM and Pap	Phase 1: 85.5% Phase 2: 54.4%	Phase 2: 49.0%
ST	Phase 1: 87.5% Phase 2: 52.4%	Phase 2: 29.7%
Test sensitivity/specificity for CIN2+		
HPV, provider collection^19^	0.78/0.89	0.67/0.86
Pap, 30-49 y^19^	0.41/0.94	0.70/0.90
Pap, >50 y^20^	0.33/0.94	0.70/0.90
Test sensitivity/specificity for CIN1+, colposcopy^3d^	0.98/0.03	1.0/1.0
Eligibility for cryotherapy, ST cohort^4,21^		
No lesion or CIN1	90%	75%
CIN2	85%	60%
CIN3	75%	49%
Cancer	10%	10%
Effectiveness of cryotherapy, ST cohort^20,22,23e^	88%	75%
Proportion of women retaining an HPV infection following cryotherapy, ST cohort	15%	30%
Effectiveness of treatment with cryotherapy or LEEP following colposcopy^23e^	94%	85%
Proportion of women retaining an HPV infection following colposcopic diagnosis and treatment	10%	30%
Direct medical costs (US$)^5^		
HPV test (clinic)^f^	$7.10	$9.60-$17.10
Pap (clinic)	$4.54	50%-150%
Colposcopy and biopsy (hospital)	$88.01	50%-150%
Cryotherapy (clinic or hospital)^e,g^	$9.70	50%-150%
LEEP (hospital)^e^	$45.79	50%-150%
Simple hysterectomy^e^	$813.97	
Pap (hospital; follow-up after treatment at hospital)	$3.99	50%-150%
Direct non-medical costs (US$)^5,17h^		50%-150%
Transportation (round-trip, clinic)	$0.76	
Transportation (round-trip, hospital)	$3.05	
Transportation (round-trip, cancer center)	$8.14	
Women's time costs (US$)		
Screening	$5.23	
Cryotherapy (clinic, ST cohort)	$4.12	
Colposcopy	$8.98	
Cryotherapy (hospital)	$8.60	
LEEP	$8.98	
Simple hysterectomy	$53.97	
Treatment of local cancer (US$) (FIGO stages 1a-2a)	$4570	50%-150%
Treatment of regional/distant cancer (US$) (FIGO stages ≥2b)	$5481	50%-150%

Abbreviations: CIN, cervical intraepithelial neoplasia; CIN1+, cervical intraepithelial neoplasia grade 1 or higher; CIN2+, cervical intraepithelial neoplasia grade 2 or higher; CM, colposcopy management cohort; FIGO, Federation Internationale de Gynecologies et Obstetriques; LEEP, loop electrosurgical excision procedure; ST, screen and treat cohort; US$, 2014 United States dollars.

a Phase 1 and phase 2 results were compared in the baseline analysis, with all variables equivalent except for compliance variables and age-specific prevalence of high-risk HPV. Cost data were originally collected in 2012 US$ (with the exception of the HPV test), and were converted to 2014 US$ using El Salvador gross domestic product deflators. The sensiti vity analysis ranges for cost data were applied to the original 2012 US$.

b Compliance for Pap was assumed to be the same as CM. In sensitivity analyses, we examined the impact of compliance with each visit at the lower bound of the 95% confidence intervals indicated by the study. Because all women in the study received their HPV results, it was assumed in sensitivity analysis that only 90% received results.

c In the ST cohort, compliance with colposcopy and treatment was only relevant for women who were determined to be ineligible for cryotherapy at the clinic.

d Test performance characteristics of colposcopy were based on the worst diagnosis of the local pathologist relative to the gold standard (i.e., worst diagnosis by a quality control pathologist), at a treatment threshold of CIN1+.

e For ST, cryotherapy was assumed to occur at the clinic for eligible women. For women requiring treatment after colposcopy (i.e., women in CM diagnosed with CIN1+; women in ST deemed ineligible for cryotherapy at the clinic), treatment with cryotherapy, LEEP, or simple hysterectomy was assumed to occur at the hospital as follows (based on study data): <CIN2, 99.6% cryotherapy, 0.3% LEEP, 0.1% simple hysterectomy; CIN2: 92.7% cryotherapy, 4.5% LEEP, 2.7% simple hysterectomy; CIN3: 53.4% cryotherapy, 28.6% LEEP, 18% hysterectomy.4 The effectiveness of cryotherapy includes management of residual disease detected during follow-up. It was assumed that women receiving cryotherapy or LEEP would receive follow-up including a Pap test at the clinic (for ST) or hospital (for CM or ST deemed ineligible for immediate cryotherapy) and a colposcopy in the year following treatment, with 1% of women receiving an additional Pap following a positive colposcopy result.

f This includes the cost of the HPV test, which was assumed to be 2014 US$5.

g On average, it was assumed 30 women could be treated per US$286 nitrous oxide tank refill.

h Details regarding the valuation of women's time and transportation have been previously described.^5^

HPV testing with provider collection of HPV specimens was assumed to take place every 5 years between ages 30 and 65 years (colposcopy management and screen and treat), while Pap testing with colposcopy management was assumed to take place every 2 years between ages 20 and 65 years (consistent with recommended screening ages in national guidelines).

In accordance with guidelines for cost-effectiveness analysis, a societal perspective was applied, including costs irrespective of the payor.^15^ Cost data are presented in Table 1. Direct medical cost data were estimated in phase 1 using a microcosting methodology.^5^ For phase 2, all costs were updated from 2012 to 2014 US$ using gross domestic product (GDP) deflators;^16^ cost per cryotherapy was updated to reflect the cost per nitrous oxide tank refill and average number of patients treated per tank in phase 2; and the cost of fuel used to transport HPV and Pap specimens to the laboratory was added. Women's time spent traveling, waiting for, and receiving care was valued using national household income data; women's transportation costs to travel to healthcare facilities were estimated by in-country clinicians.^5,17^

Reported model outcomes include lifetime risk of cervical cancer, expected total lifetime cost per woman, and life expectancy. After discounting future costs and life-years at a rate of 3% per year, incremental cost-effectiveness ratios (ICERs) were calculated. ICERs represent the additional cost of a strategy divided by its additional benefit relative to the next most costly strategy after eliminating strategies that are either more costly and less effective, or have higher ICERs than more effective strategies (i.e., dominated). A “very cost-effective” intervention was defined as one with an ICER (in US$ per year of life saved [YLS]) less than El Salvador's per capita GDP of US$3990.^18^

Sensitivity analyses were conducted to examine the impact of alternative values for model inputs (Table 1). Additionally, the analysis was repeated using visit compliance data from phase 1 to directly compare cost-effectiveness results for phase 1 with phase 2 using the updated model and holding other input parameters constant.

Ethics approval was not required owing to the retrospective design, as de-identified data were analyzed retrospectively as part of a previous analysis. All procedures conducted as part of CAPE were approved by the national ethics review board of El Salvador.

## 3 RESULTS

In phase 2, HPV testing with screen and treat was the most effective screening strategy, predicted to reduce the absolute risk of cervical cancer by 58.5% (Table 2). Pap with colposcopy management was substantially less effective (primarily due to lower test sensitivity for precancer and greater number of visits required for diagnosis and treatment) reducing cancer risk by 43.6%. Although colposcopy management and Pap were assumed to have the same visit compliance rates and colposcopy management utilized HPV testing, colposcopy management was the least effective strategy, predicted to reduce cancer risk by 41.7%. This was due to the later start age at screening with HPV-based screening relative to Pap (i.e., 30 years rather than 20 years) in this analysis.

**TABLE 2:** Cost, health, and cost-effectiveness outcomes in phase 1 vs phase 2 (El Salvador per capita GDP: US$3990).^a^

Screening strategy	Reduction in lifetime risk of cervical cancer (%)^b^	Discounted lifetime cost per woman (US$)	Discounted life expectancy (years)	ICER (US $/YLS)
Phase 1^c^				
No screening	-	35.40	28.86490	
ST	62.2	76.46	28.95188	470
CM	57.4	90.45	28.94526	Dom
Pap	58.2	222.31	28.95730	26 900
Phase 2				
No screening	-	35.40	28.86490	
ST	58.5	74.58	28.94422	490
CM	41.7	86.47	28.92491	Dom
Pap	43.6	203.17	28.93703	Dom

Abbreviations: GDP, gross domestic product; CM, colposcopy management cohort; Dom, dominated strategy; ICER, incremental cost-effectiveness ratio; ST, screen and treat cohort; US$, 2014 United States dollars; YLS, year of life saved.

a For reduction in cancer risk, discounted lifetime costs, and discounted life expectancy from age 9 years, the mean value is reported across 50 input parameter sets; the reported ICER is the ratio of the mean costs divided by the mean effects of one strategy vs another across the 50 sets.

b Relative to no screening.

c Phase 1 results are different than previously published estimates5 due to updates in the natural history model and calibration, updated test performance data, updating of costs to 2014 US$ and updated fuel and cryotherapy gas costs, and the start age for Pap screening beginning at age 20 y (rather than age 30 y, in the previous analysis). Phase 1 and 2 results differ only in visit compliance parameters, as indicated in Table 1.

Because screen and treat was both less costly and more effective than colposcopy management and Pap, it was the dominant strategy, with an ICER of US$490 per YLS, compared with no screening. Given El Salvador's per capita GDP of US$3990, screen and treat would be considered very cost-effective in El Salvador.

**Figure 1:**
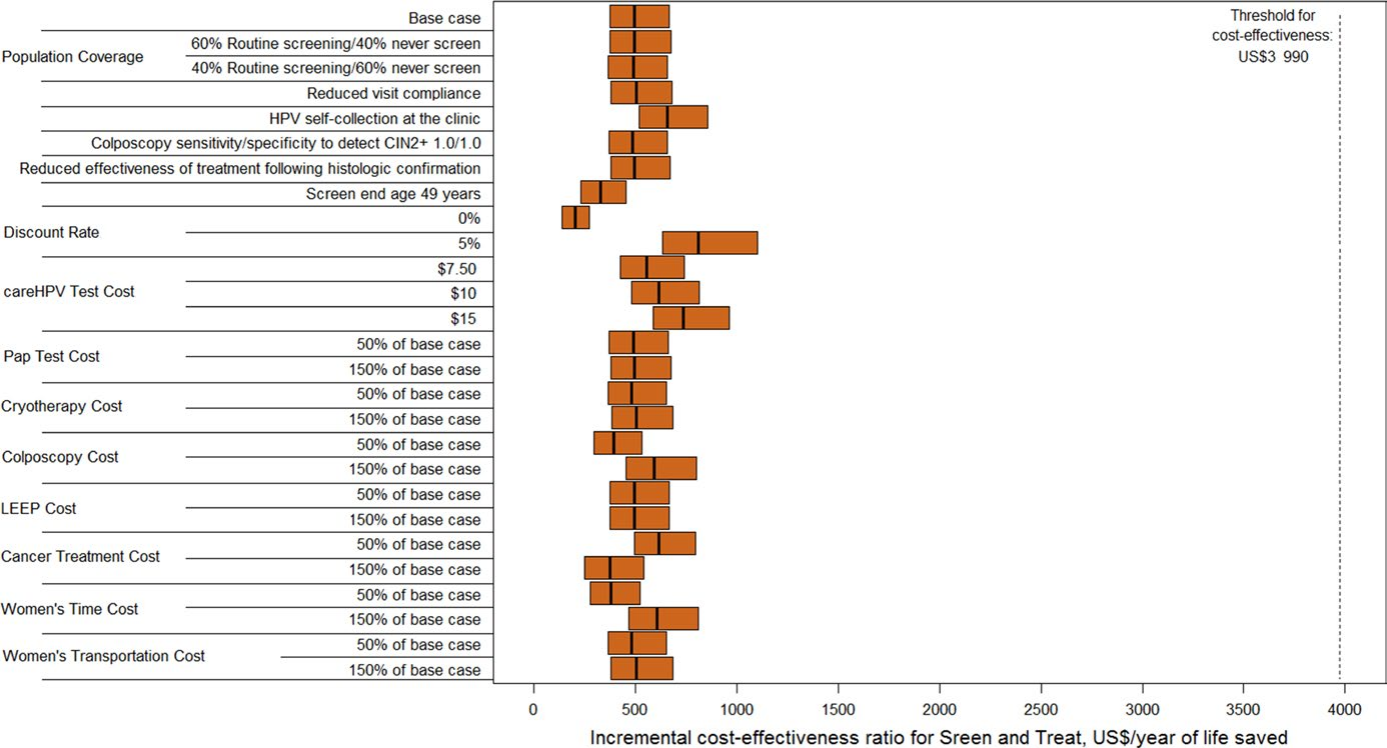
Cost-effectiveness analysis: Base case and sensitivity analysis. Abbreviation: LEEP, loop electrosurgical excision procedure. Incremental cost-effectiveness ratios (ICERs) for the phase 2 screen and treat (ST) strategy are presented along the x-axis in 2014 US$ per year of life saved for the base case analysis and sensitivity analyses (y-axis). The orange bars represent the range of the ICERs for ST (dominant strategy) across the 50 input parameter sets, with the ICER of the mean costs divided by the mean effects demarcated by a black line. The dashed black line indicates El Salvador's per capita gross domestic product (GDP) at US$3990, which was considered as a threshold for identifying very cost-effective interventions.

Phase 2 results were robust across a wide range of sensitivity analyses (Fig. 1). Screen and treat remained the dominant strategy and very cost-effective as the following scenarios were explored: reduction in screening coverage of the target population; reduction in visit compliance; reduction in HPV test sensitivity (to resemble self-collection of HPV specimens with careHPV); improved test performance of colposcopy, screening end age of 49 years; reduction and increase in the discount rate; increased cost of the HPV test; and reduction or increase in the costs of Pap, colposcopy, LEEP, cancer treatment, and women's time and transportation.

In several sensitivity analyses, screen and treat no longer dominated Pap but still maintained the lowest ICER. When Pap test sensitivity to detect CIN2+ improved, screen and treat had an ICER of US$490/YLS, while Pap had an ICER of US$12 480. When eligibility for cryotherapy was reduced, the ICERs for screen and treat and Pap were US$570 per YLS and US$461 490 per YLS, respectively. When the effectiveness of cryotherapy was reduced, the ICERs for screen and treat and Pap were US$580 per YLS and US$348 470 per YLS, respectively. Though Pap was slightly more effective than screen and treat in these instances, the ICER for Pap was prohibitively expensive. Thus, screen and treat remained the most effective strategy with an ICER below per capita GDP in all sensitivity analyses.

Results comparing phases 1 and 2—using the updated model, costs, and input parameters, except for phase-specific visit compliance indicators—are presented in Table 2. Due to slightly higher visit compliance in the phase 1 screen and treat cohort, screen and treat was slightly more effective and had a lower ICER in phase 1 (US$470 per YLS vs US$490 per YLS in phase 2). Although Pap yielded lower reductions in cancer risk relative to screen and treat in phase 1, it was associated with slightly greater life expectancy gains because it detected a small number of early cancers between ages 20 and 30 years. However, these gains were very costly and yielded an ICER of US$26 900.

## 4 DISCUSSION

In the present study, a microsimulation model of HPV infection and cervical cancer was updated to fit epidemiologic data from phase 2 of the CAPE project, in which HPV-based cervical cancer screening is being implemented in El Salvador's public sector. The cost-effectiveness of two management algorithms (colposcopy management and screen and treat) for HPV-positive women was estimated relative to Pap screening using phase 2 data for costs, visit compliance, and eligibility for cryotherapy. The present study found that HPV testing followed by immediate treatment with cryotherapy for all eligible women (screen and treat) every 5 years between ages 30 and 65 years would be very cost-effective in El Salvador, reducing the risk of cervical cancer by nearly 60% and costing US$490 per YLS. Across all sensitivity analyses, screen and treat was consistently the most effective screening strategy with an ICER below the cost-effectiveness benchmark of El Salvador's per capita GDP.

The cost-effectiveness of screening strategies using phase 1 data on visit compliance to provide updated estimates for direct comparison was reanalyzed. Reassuringly, the slightly diminished compliance with recommended follow-up seen in phase 2 had little impact on the reduction in cancer risk or cost-effectiveness of screen and treat. The vast majority of eligible women in the screen and treat cohort received immediate cryotherapy in both phase 1 and phase 2; compliance with colposcopy and treatment was lower among women ineligible for immediate cryotherapy in phase 2. As scale-up of screen and treat continues, ensuring timely follow-up for ineligible women will remain a priority.

The phase 2 ICER for screen and treat in the present analysis (US$490 per YLS) was lower than the previously published phase 1 estimate (US$2040 per YLS) for several reasons.^5^ The present analysis used an updated model with greater flexibility to adapt to settings with epidemiologic diversity, reflecting greater uncertainty in natural immunity and the progression and regression of precancer. The updated model fitted projected cervical cancer incidence in El Salvador well,^1^ while the previous model underestimated cancer incidence. This underestimation of cervical cancer incidence contributed to higher ICERs in the earlier analysis. In the present analysis of phase 1, Pap was associated with more life-years gained than screen and treat because it was assumed Pap started at age 20 years, while the previous analysis assumed all screening strategies started at age 30 years. In actuality, HPV-based strategies would likely be preceded by Pap testing for some women under 30 years, and followed by Pap testing in women over age 59 years, in accordance with national guidelines that were recently amended to reflect findings from CAPE.

Several limitations should be noted. First, programmatic cost data to reflect MINSAL's investment in information systems for creating a national screening registry were not available, nor were patient outreach costs included. Second, screening was evaluated for ages 30–65 years (HPV testing) and 20-65 years (Pap), but phase 2 only involved screening for ages 30-49 years. Thus, HPV prevalence data at older and younger ages was unavailable for model calibration, and the model may have under- or overestimated the benefits of screening outside the age range of 30–49 years. Model fit to cancer incidence data was also based on GLOBOCAN projections, because El Salvador does not have a national cancer registry. National guidelines regarding recommended screening ages and follow-up of treated women are being reconsidered; thus, the assumption in the present study that women receive a Pap test and colposcopy in the year following treatment may be in flux. Finally, there was lack of consensus around how to define a cost-effectiveness threshold. The WHO Commission on Macroeconomics and Health is often cited as the source of the per capita GDP benchmark,^18^ yet recent analyses suggest that using per capita GDP as a threshold for willingness to pay may displace interventions yielding substantial health benefits in favor of less effective interventions.^24^ Because the ICER for screen and treat is well below 50% of per capita GDP, it is likely that the strategy would remain “cost-effective” even at a lower threshold.

Phase 3 of the CAPE project, commenced in May 2015, is currently underway with the goal of screening 20 000 women per year with screen and treat to reach 80% of screening-eligible women within 5 years. As scale-up continues, health promotors will target underscreened women, potentially reaching women at greater risk of cervical cancer. Cryotherapy will not be available at all screening facilities and, in some cases, screen-positive women will be referred to receive treatment elsewhere. Continued follow-up of treated women in phases 1 and 2 will also provide data on cryotherapy effectiveness. As further data on implementation costs and health impact become available, cost-effectiveness estimates will continue to be updated to provide decision makers with information on one of the first national HPV-based screening programs in a lower-middle-income country.

## Supplementary Material

Figure S1 Model fit, 50 input parameter sets: Prevalence of oncogenic HPV, phase 2

Figure S2 Model fit, 50 input parameter sets: Prevalence of HPV16/18 in cervical cancer

Figure S3 Model fit, 50 input parameter sets: Cervical cancer incidence
