# Supplementary figures and images for: The cost-effectiveness of implementing HPV testing for cervical cancer screening in El Salvador
Source: Int J Gynaecol Obstet. 2019 Jan 31;145(1):10.1002/ijgo.12773. doi: 10.1002/ijgo.12773 (PMC6988124; doi:10.1002/ijgo.12773)

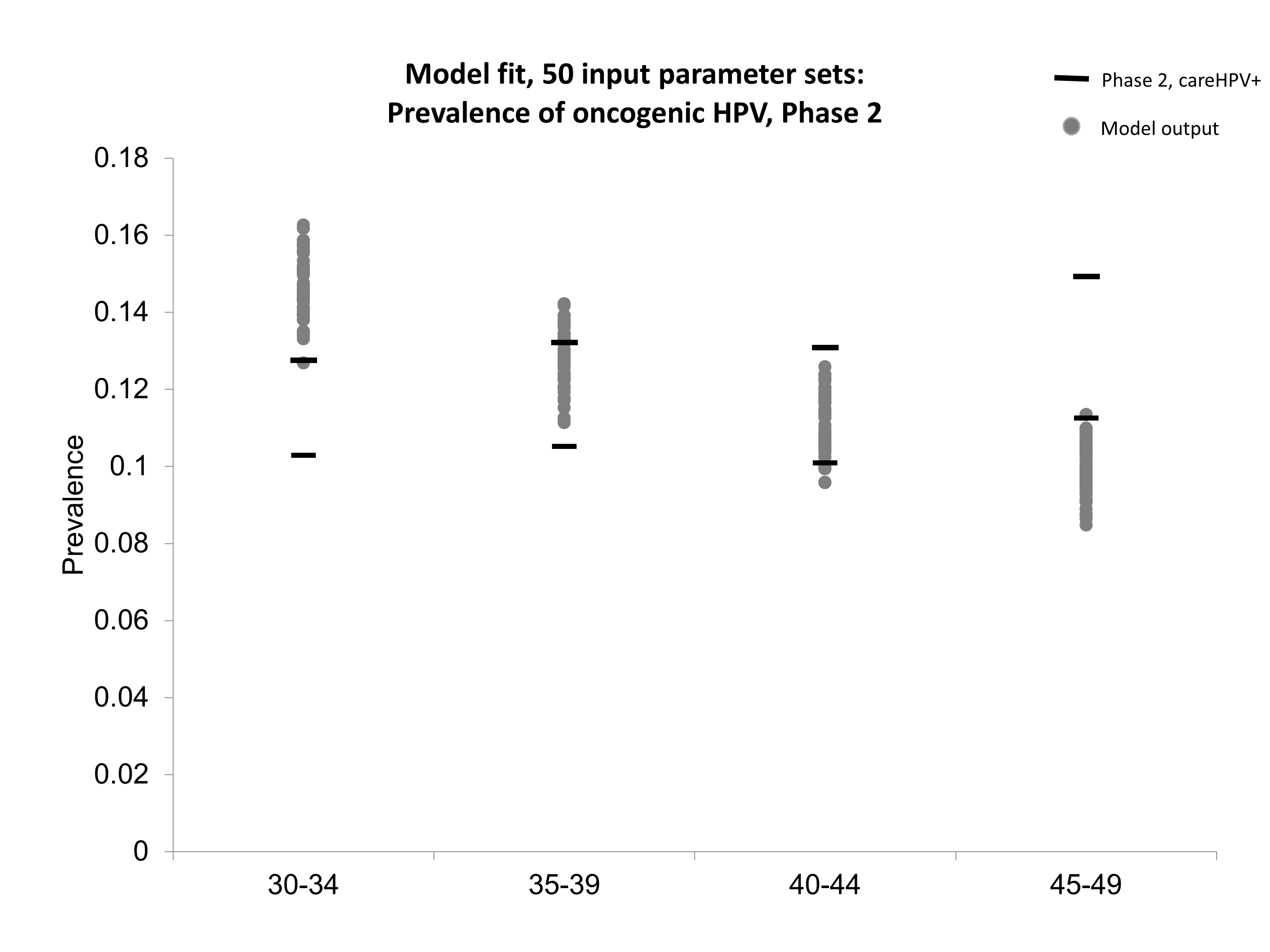

Supplement: Figure S1 [file ijgo-2019-12773-s1.tif]

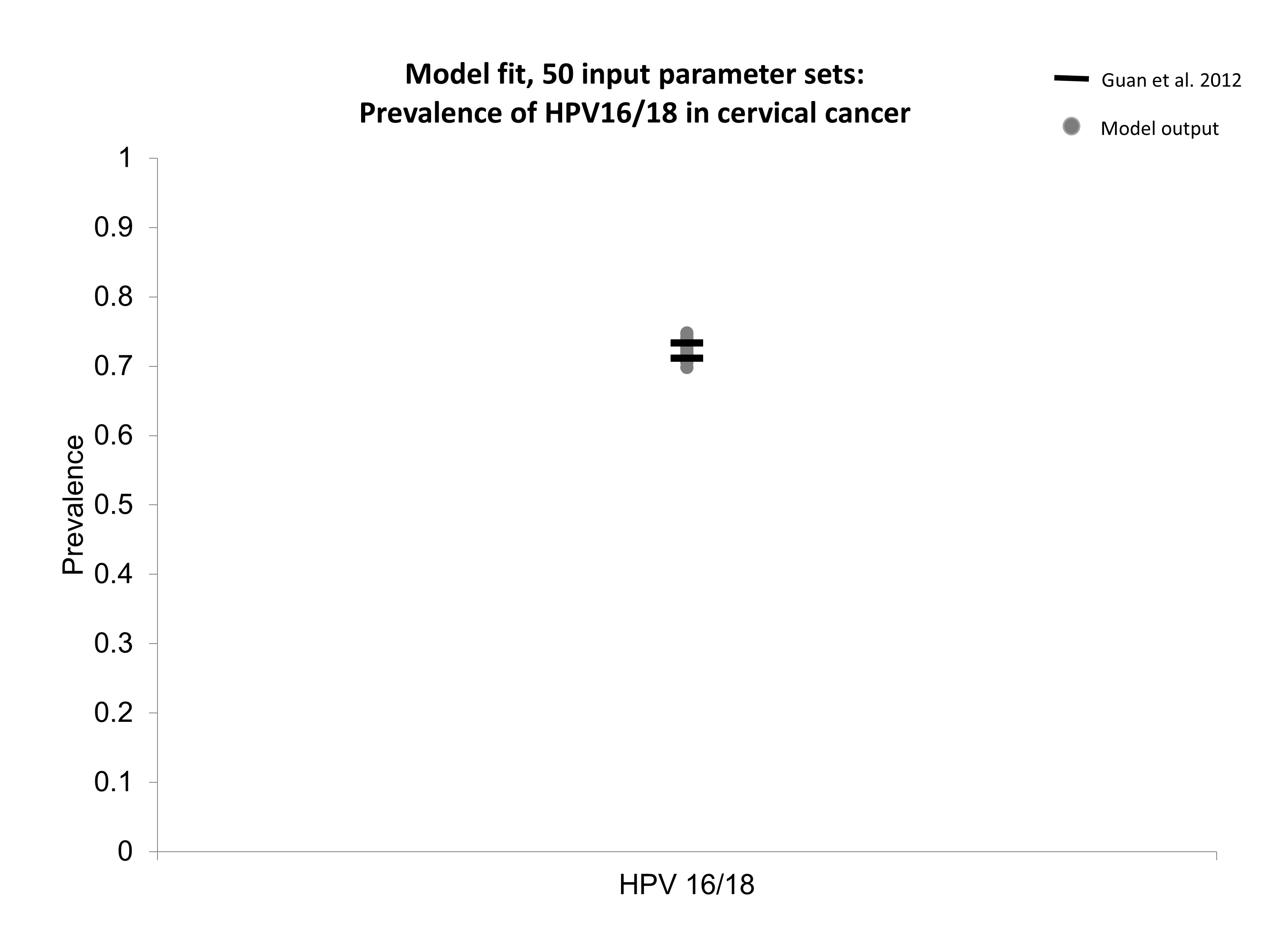

Supplement: Figure S2 [file ijgo-2019-12773-s2.tif]

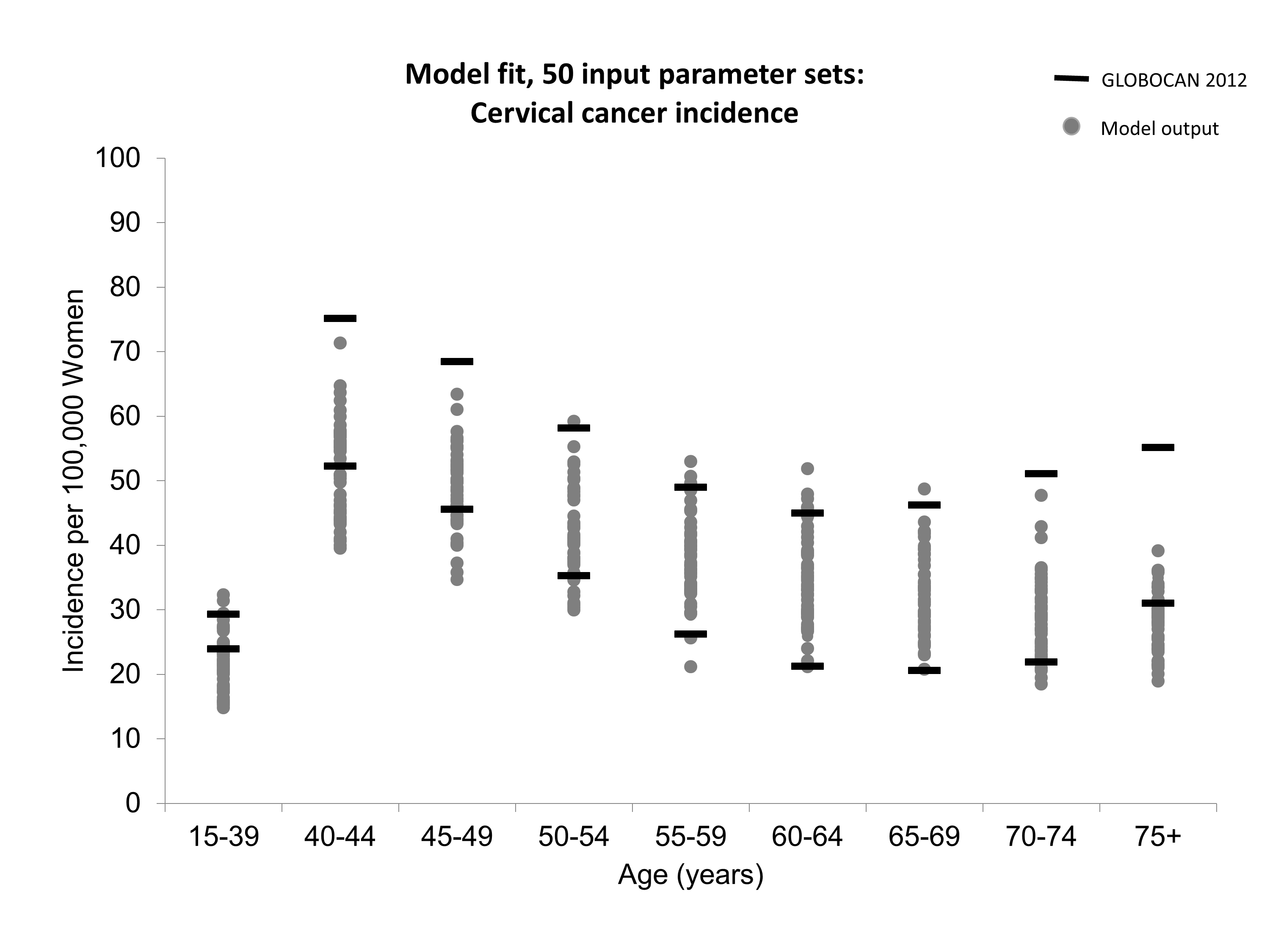

Supplement: Figure S3 [file ijgo-2019-12773-s3.tif]
